# Biochemical and proteomics analyses of antioxidant enzymes reveal the potential stress tolerance in *Rhododendron chrysanthum* Pall

**DOI:** 10.1186/s13062-017-0181-6

**Published:** 2017-05-03

**Authors:** Xiaofu Zhou, Silin Chen, Hui Wu, Yi Yang, Hongwei Xu

**Affiliations:** Jilin Provincial Key Laboratory of Plant Resource Science and Green Production, Jilin Normal University, Siping, 136000 China

**Keywords:** *Rhododendron chrysanthum* Pall., Proteomics, Antioxidant enzymes, Stress tolerance

## Abstract

**Background:**

*Rhododendron chrysanthum* Pall., an endangered species with significant ornamental and medicinal value, is endemic to the Changbai Mountain of China and can also serve as a significant plant resource for investigating the stress tolerance in plants. Proteomics is an effective analytical tool that provides significant information about plant metabolism and gene expression. However, no proteomics data have been reported for *R. chrysanthum* previously. In alpine tundra, the abiotic stress will lead to a severe over-accumulation of reactive oxygen species (ROS). Many alpine plants overcome the severe stresses and protect themselves from the oxidative damage by increasing the ratio and activity of antioxidant enzymes.

**Results:**

In our study, wild type and domesticated *Rhododendron chrysanthum* Pall. were used as experimental and control groups, respectively. Proteomics method combined with biochemical approach were applied for the stress tolerance investigation of *R. chrysanthum* at both protein and molecular level. A total of 1,395 proteins were identified, among which 137 proteins were up-regulate in the experimental group. The activities of superoxide dismutase (SOD), catalase (CAT), ascorbate peroxidases (APXs), and glutathione peroxidase (GPX) were significantly higher and the expression of APXs and GPX were also increased in the experimental group. Moreover, the interaction network analysis of these enzymes also reveals that the antioxidant enzymes play important roles in the stress resistance in plants.

**Conclusions:**

This is the first report of the proteome of *Rhododendron chrysanthum* Pall., and the data reinforce the notion that the antioxidant system plays a significant role in plant stress survival. Our results also verified that *R. chrysanthum* is highly resistant to abiotic stress and can serve as a significant resource for investigating stress tolerance in plants.

**Reviewers:**

This article was reviewed by George V. (Yura) Shpakovski and Ramanathan Sowdhamini.

**Electronic supplementary material:**

The online version of this article (doi:10.1186/s13062-017-0181-6) contains supplementary material, which is available to authorized users.

## Background

*Rhododendron chrysanthum* Pall. (*R. chrysanthum*), belonging to the family of *Ericaceae*, is one of the most precious germplasm resources in the world. In China, *R. chrysanthum* only grows at altitudes between 1,300 m and 2,650 m on the Changbai Mountain where belongs to alpine tundra. Changbai Mountain is a hibernating volcano located at the junction of China and North Korea and was formed during the Quaternary glacial period [1]. At the top of the mountain, the annual average temperature is -7.3 °C. The harsh climate and poor soil at the top of the Changbai Mountain are serious challenges for plants. The long adaptive evolution process of *R. chrysanthum* evolved resistance to the cold temperatures, drought, strong UV radiation and other abiotic stresses.

Proteomics was defined and proposed in 1995 [2]. Nowadays, with the development of the omics, the technique of proteomics has already become an effective tool for understanding plants at the proteomics level [3]. Proteomics approaches have been widely used in studies of plant growth and development [4], secondary metabolism [5], cell death [6] and stress tolerance [7]. In the field of plants stress resistance, especially in abiotic stress, proteomics has made a tremendous contribution [8]. Proteomics studies in the category of abiotic stress have been focused mainly on cold temperatures [9], drought [10], flooding [11], salinity [12] and heavy metals [13]. To date, the plant materials used in proteomics researches including rice, wheat, beans and many other plants [14]. However, the technique of proteomics has not been used widely in the study of alpine plants [15].

In previous studies of alpine plants, morphological, physiological and biochemical approaches were used to understand the plants’ underlying molecular and physiological mechanisms for adapting to tough environments. The results of these studies showed that, to face the harsh circumstances of high altitudes, alpine plants have evolved through changes in many different features at morphological and physiological levels [16].

In the present study, TMT labeling integrated with LC-MS/MS was used to quantify the dynamic changes of the whole proteome of *R. chrysanthum*. Furthermore, the proteomics methods were combined with biochemical analysis to unravel the contribution of superoxide dismutase (SOD), catalase (CAT), ascorbate peroxidases (APXs) and glutathione peroxidase (GPX) to the stress resistance of *R. chrysanthum*. These results provide the first proteome-wide view of the *R. chrysanthum* and reinforce the notion that the antioxidant system plays a significant role in the environment adaptation and the stress tolerance of plants.

## Results

### Proteome-wide analysis of *Rhododendron chrysanthum* Pall.

**Fig. 1:**
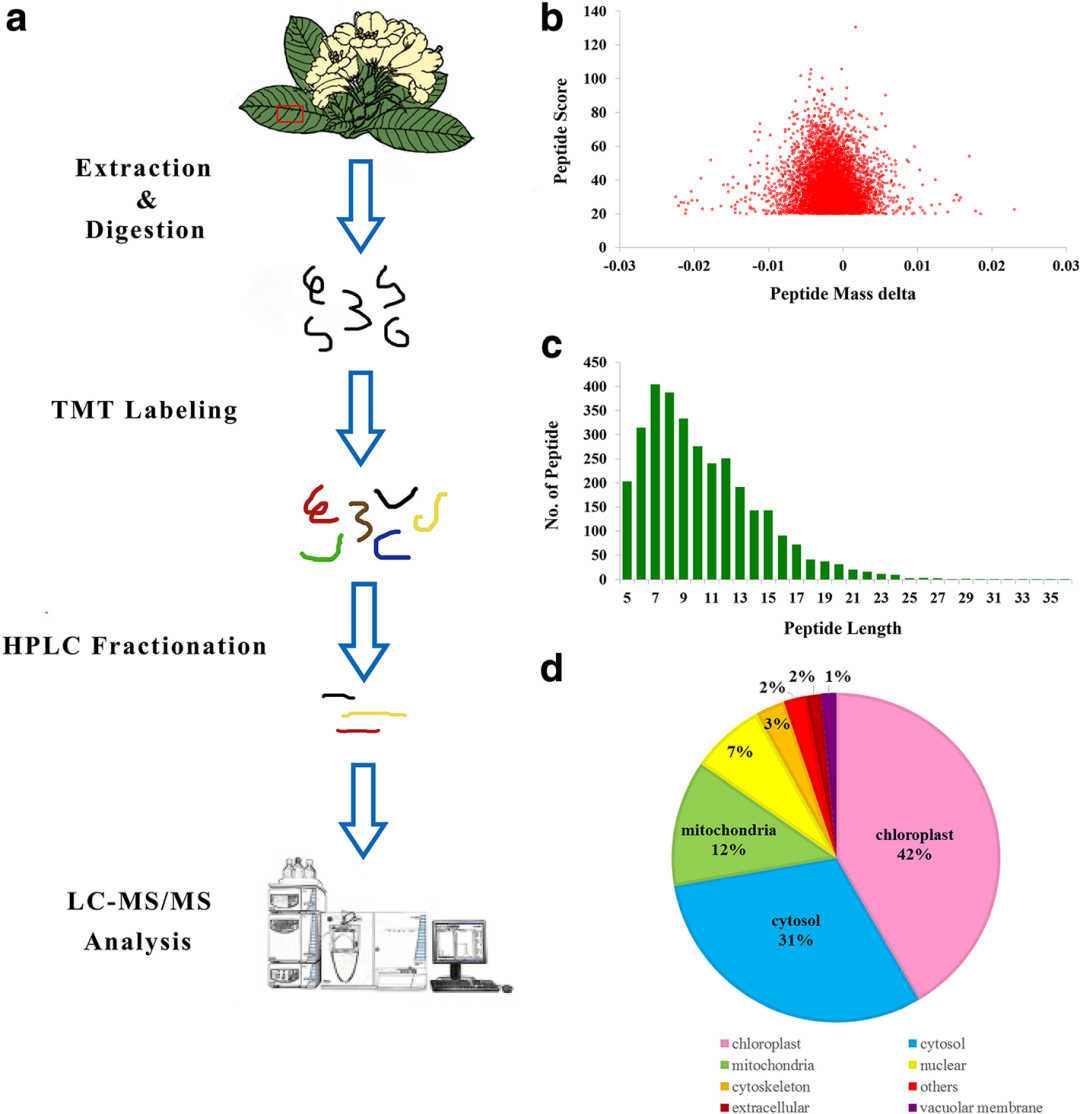
Proteome-wide analysis of *Rhododendron chrysanthum* Pall. **a** Overview of experimental process of this study. **b** Mass error distribution of all identified peptides. **c** Peptide length distribution. **d** The subcellular location of up-regulated proteins in experimental group

The overview of experimental design is shown in Fig. 1a. Briefly, proteins were first extracted and digested into peptides. TMT labeling and LC- MS/MS were then used to analyze and quantify the dynamic changes of the proteome. The distribution of mass error is near zero and most of peptides are less than 0.02 Da (Fig. 1b). The length of most peptides distributed between 8 and 16, which agreed with the properties of the tryptic peptides (Fig. 1c). In the present work, 1,395 proteins were identified and 705 of those proteins were quantified. A quantitative ratio over 1.3 was considered as up-regulation (UR). Based on this standard, 137 up-regulated proteins were identified in the experimental group (EG), compared with the control group (CG). A Gene Ontology (GO) functional classification was then used to further understand the whole UR protein distribution in the EG. Most proteins were involved in metabolic processes, cellular processes, and cell and membrane physiology. Under the category of molecular function, we identified 14 UR proteins with antioxidant activity. The results of subcellular localization analysis showed that the UR proteins were mainly localized in chloroplast (42%), cytosol (31%), and mitochondria (12%) (Fig. 1d). These data suggested that the changes in *R. chrysanthum* covered a broad range of cellular processes and most were localized in crucial cellular compartments that play important roles in plant development.

### Analysis of superoxide dismutase and related proteins

**Fig. 2:**
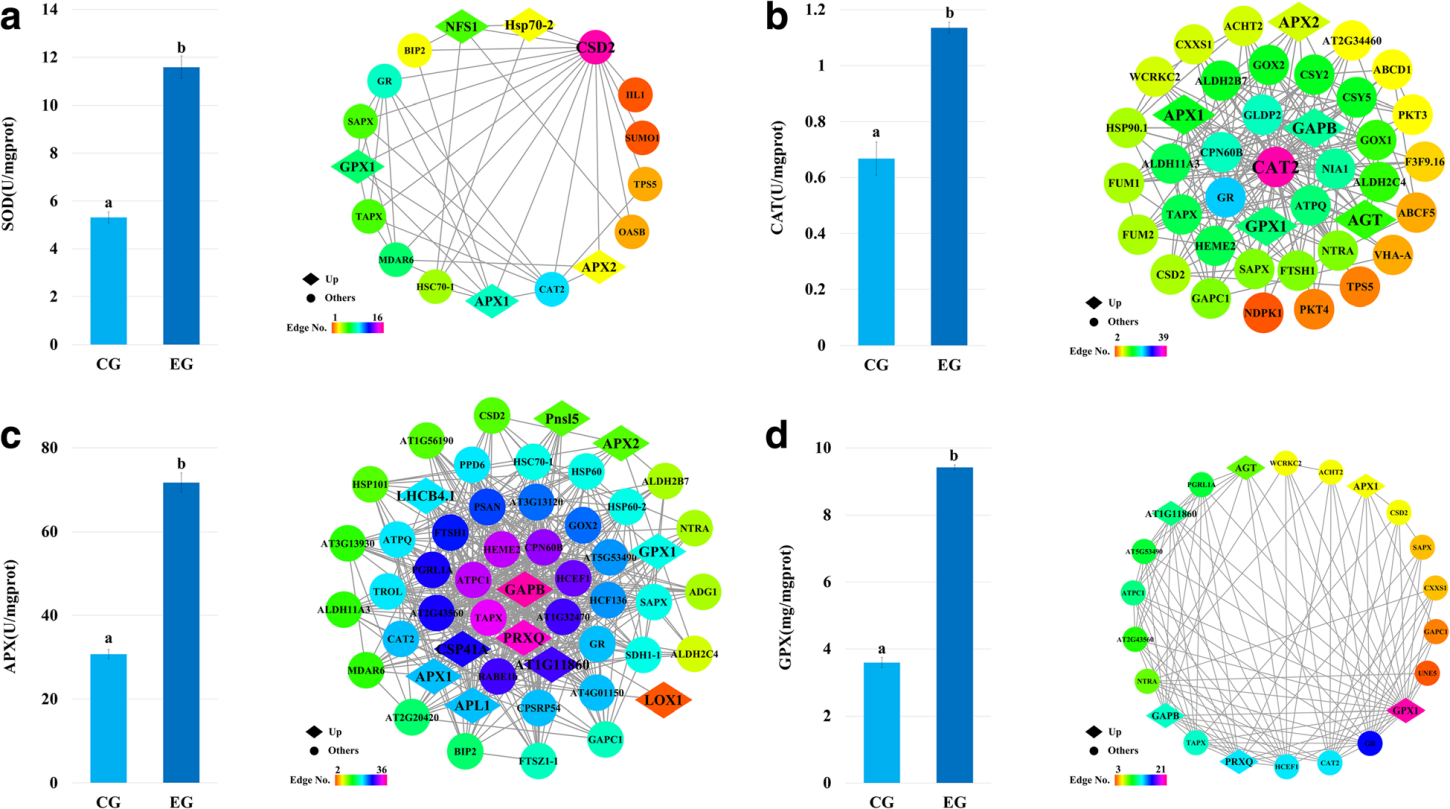
Activity analyses and the interaction networks of SOD **a**, CAT **b**, APX **c** and GPX **d** SOD refers to superoxide dismutase, CAT refers to catalase, APX refers to ascorbate peroxidases and GPX refers to glutathione peroxidases. EG and CG stand for the experimental group and the control group, respectively. Values are expressed as means ± SD, *n* = 3. Statistically different values (*p* < 0.05) are indicated by different letters

Superoxide dismutase (SOD, EC 1.15.1.1) are the first line enzymes that protect plants from ROS damage; they convert O^2•−^ into H_2_O_2_ [17]. Three types of SOD (Fe-SOD, Mn-SOD, and Cu/Zn-SOD) are located in different organelles. The Cu/Zn-SOD included two types: CSD-1 and CSD-2 (CSD), which were both located in the chloroplast [18]. The CSD-2 was quantified in present study. All quantified proteins (705) were used as background and the proteins related to CSD-2 were identified using String and Cytoscape software. The network of protein interactions is shown in Fig. 2a. In total, 16 proteins had direct interaction with CSD-2. Among these, 5 proteins were up-regulated in this network. The activity of SOD was assayed and analyzed at the same time (Fig. 2a). The activity of SOD was increased rapidly in the EG, accounting for approximately 118% increase of CG value.

### Analysis of catalase and related proteins

Catalase (CAT, E.C.1.11.1.6), an iron porphyrin enzyme which mainly localized in peroxisomes, can effectively remove the H_2_O_2_ and prevent the over-accumulation of reactive oxygen species (ROS) [19]. The types of CAT are varied in different plants and in recent study three types of CAT (CAT1, CAT2 and CAT3) were quantified in *R. chrysanthum*. However, only CAT2 was involved in the network that used total quantified proteins as the background and obtained from String (Fig. 2b). The network of CAT2 was made up of 40 proteins, including 5 up-regulated proteins (GAPB, GPX1, AGT, APX1, and APX2). At the same time, the determination of CAT activity revealed significantly higher activity in the EG than in the CG, or an increase of nearly 70% (Fig. 2b).

### Analysis of ascorbate peroxidases and related proteins

Ascorbate peroxidases (APXs, EC 1.11.1.1) are the key enzymes of hydrogen peroxide detoxification system that can convert H_2_O_2_ into water. The isoforms of APX were classified based on their subcellular localization [20]. The protein-protein interaction network designed by String and visualized by Cytoscape is shown in Fig. 2c, where the color represents the weight of each protein in the network. Four types of APX, including APX1, APX2, SAPX (localized in chloroplast stroma), and TAPX (localized in chloroplast thylakoids), were identified in the network. Overall, 52 proteins (including four major APXs) were involved in this interaction network. Among them, 11 proteins containing APX1 and APX2 were expressed at much higher levels in the EG compared with CG. In present work, the activity of APX in EG was significant higher compared with CG, an increase of 133% (Fig. 2c).

### Analysis of glutathione peroxidase and related proteins

Glutathione peroxidase (GPX, EC 1.11.1.9) are efficient ROS scavengers with high affinity for H_2_O_2_ [21]. They can reduce the H_2_O_2_ and organic hydroperoxides, thereby protecting cells from oxidative damage [22]. GPXs were widely distributed in plant cells with highly conservative cysteine residue. Eight GPXs in Arabidopsis were found in previous studies [23]. In our latest study, we identified two types of GPX (GPX1 and GPX7) in *R. chrysanthum*. However, only GPX1 can be retrieved in the String database and 21 relevant proteins were found among the total quantified proteins (705). The network of GPX1 and related proteins is shown in Fig. 2d. Overall, 6 proteins containing GPX1, the core protein of this network, were upregulated. Apart from GPX1, the expression of APX1, another important enzyme in H_2_O_2_ scavenging, was also increased in this network. The GPX antioxidant activity increased sharply in the EG, which was approximately 262% of the activity in the CG (Fig. 2d).

### Antioxidant protein interaction network in *Rhododendron chrysanthum* Pall.

**Fig. 3:**
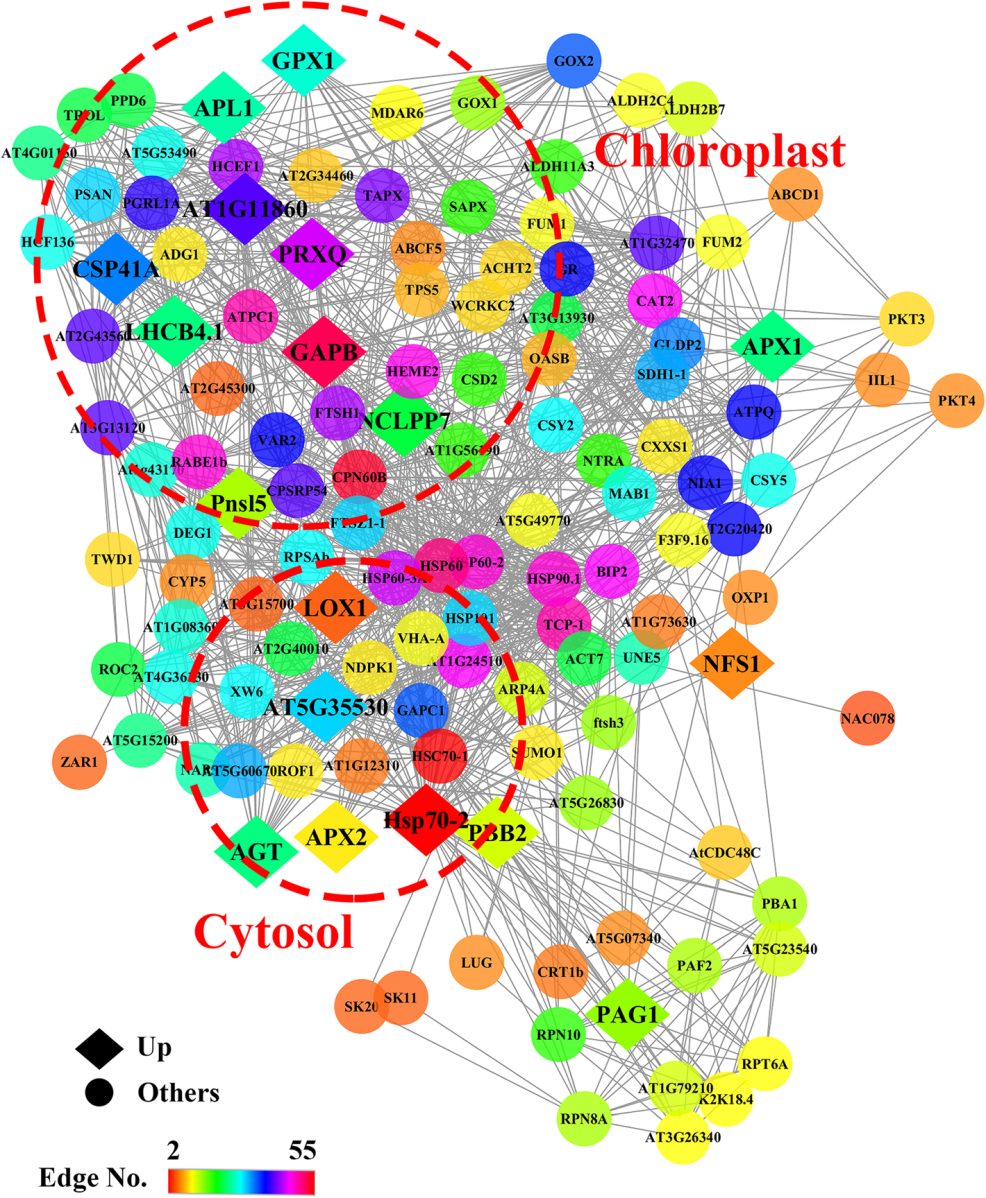
Interaction network of SOD, CAT, APX, GPX and their related proteins in *Rhododendron chrysanthum* Pall.

**Fig. 4:**
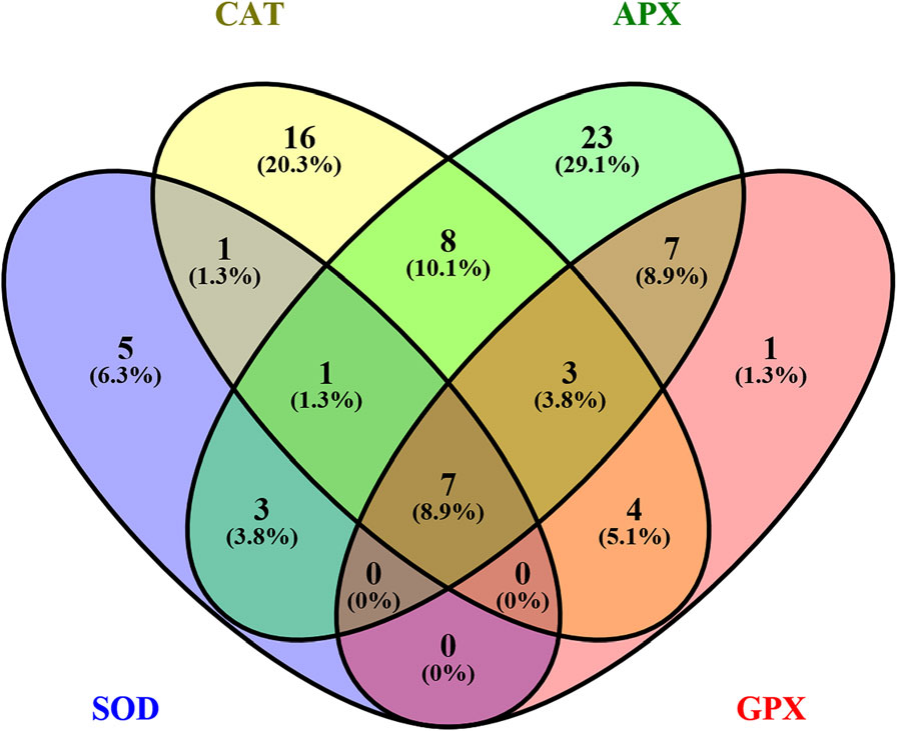
Venn diagram for SOD, CAT, APX, GPX and their related proteins in *Rhododendron chrysanthum* Pall.

The role of the UR proteins in this alpine plant was investigated by setting up the protein interaction networks of mainly antioxidant proteins—SOD (CSD2), APX (APX1, APX2, SAPX, and TAPX), CAT (CAT2), and GPX (GPX1)—via String and Cytoscape (Fig. 3). Overall, 129 proteins were mapped in the present study, including 7 main proteins (CSD2, APX1, APX2, SAPX, TAPX, CAT2 and GPX1) and 122 relevant proteins. All the UR proteins in the networks were shaped as a rhombus. The connectedness and weights of the proteins in this network were distinguished by color. Two highly interrelated clusters of these proteins were obtained according to the Cytoscape software program. Proteins in the largest cluster (Cluster I) were mainly localized in chloroplast, and the second-largest cluster (Cluster II) consisted of proteins localized in cytosol. The Venn diagram in Fig. 4 shows the number, percentage, and overlap of proteins involved in the interactions of the four mainly categories of enzymes. Seven common proteins, CSD2, APX1, TAPX, CAT2, SAPX, GR, and GPX1, played a significant role in these four networks. Among these, two proteins were up-regulated in the EG (APX1 and GPX1). The up-regulated proteins make indispensable contributions to the whole antioxidant system and play crucial roles in the cooperation and coordination in *R. chrysanthum*.

## Discussion

*Rhododendron chrysanthum* Pall. is a cherished plant resource throughout the world and it has been reported in a few researches. However, to date, the research of *R. chrysanthum* consists of a limited number of papers on morphological, physiological, and biochemical aspects. In our present study, the proteomics was combined with a biochemical approach to investigate *R. chrysanthum* at both the protein and molecular levels. Wild type and domesticated *R. chrysanthum* varieties were used as experimental and control groups, respectively, in the current study. We quantified a total of 705 proteins, including 137 up-regulated proteins, through an integrated approach involving TMT labeling and LC-MS/MS. These up-regulated proteins covered various biological functions and were localized in multiple subcellular structures. This finding suggests that considerable changes occurred in the two types of *R. chrysanthum* and these changes play crucial roles in a wide range of cellular processes. These data are the first report of the proteome-wide analysis in *R. chrysanthum*.

Higher plants have an innate antioxidant defense system that is sensitive to abiotic stress [24]. This highly complex and regulated system is also the reason that alpine plants can survive in changing environments. In the environment of the alpine tundra, the alpine plants should face harsh circumstances, consistent with low temperature, low oxygen content, high UV radiation, and strong wind. These factors will lead to a severe over-accumulation of ROS which have potential toxicity and can also result in oxidative damage of plants. In a rugged environment, plants defense the severe stresses by increasing the ratio and activity of antioxidant enzymes. These antioxidant enzymes, including SOD, CAT, APXs and GPX, work together to detoxify ROS and protect plants from oxidative damage. In this study, the activities of these four enzymes were significantly raised in the EG compared with the CG, suggesting that the antioxidant system has clearly changed in the wild type *R. chrysanthum* and highlighting the notion that these antioxidant enzymes play key roles in the stress tolerance of plants. The expression of APXs, GPX, and the proteins that have direct interactions with SOD and CAT were increased in the EG at the protein level. The interaction network analysis of these enzymes also reveals that these enzymes and the up-regulated proteins play important roles in the stress resistance in plants. All these findings indicated that these enzymes can protect plants from oxidative injury by scavenging free radicals, and they can enhance the ability of plants to withstand a rugged environment.

## Conclusions

Our findings provide the first extensive data of the proteome of *Rhododendron chrysanthum* Pall. and an affluent dataset for the further investigation of stress tolerance in alpine plants. It also verified that the antioxidant system of *R. chrysanthum* has been successfully enhanced in the long-term adaptive process. Our results reinforce the notion that the antioxidant system plays a significant role in plants, especially in the adaptation to and tolerance of environmental stress.

## Methods

### Plant materials and growth conditions

Wild type and domesticated *Rhododendron chrysanthum* Pall. tissue seedlings were used as the experimental group (EG) and the control group (CG), respectively. The leaves excised from four-month-old plants of the EG and the CG were immediately used for protein extraction. To ensure adequately coverage, three biological replicates of each group (i.e. six plants) were collected.

### Protein extraction

Plant materials were ground into liquid nitrogen and then transferred to 5 mL centrifuge tubes and sonicated three times on ice using a high intensity ultrasonic processor (Scientz) in lysis buffer (8 M urea, 2 mM EDTA, 10 mM DTT and 1% Protease Inhibitor Cocktail). The remaining debris were removed by centrifugation at 20,000 × g at 4 °C for 10 min. The protein in the supernatant was precipitated with cold 15% TCA at -20 °C for 4 h. After centrifugation at 4 °C for 3 min, the remaining precipitates were washed with cold acetone three times. Finally, the protein was redissolved in the buffer (8 M urea, 100 mM TEAB, pH 8.0), and the protein concentration in the supernatant was estimated with a 2-D Quant kit, according to the manufacturer’s instructions.

### Trypsin digestion

For trypsin digestion, the protein solution was reduced with 10 mM DTT for 1 h at 37 °C and alkylated by adding 20 mM IAA to the mixture for 45 min at room temperature in the dark. Subsequently, the protein samples were diluted by adding 150 mM TEAB to the urea concentration for less than 2 min. After being diluted, the protein samples were digested with trypsin at a trypsin-to-protein mass ratio of 1:50 for the first digestion for 8 h, and a trypsin-to-protein mass ratio of 1:100 for a second 4 h-digestion. Approximately 100 μg protein of each sample was digested with trypsin for the following experiments.

### Tandem mass tags (TMT) labeling

After trypsin digestion, peptide was desalted by Strata X C18 SPE column (Phenomenex) and vacuum-dried. Peptide was reconstituted in 1 M TEAB and then labeled with a 6-plex TMT kit (Thermo) according to the manufacturer's instructions. Each TMT reagent was thawed and reconstituted in 24 μl acetonitrile (ACN). Finally, the peptide mixtures were incubated for 2 h at room temperature and then lyophilized by vacuum centrifugation.

### HPLC fractionation

After TMT labeling, the samples were injected into an Agilent 300 Extend C18 column (5 μm particles, 4.6 mm ID, 250 mm length) and fractionated by high pH reverse-phase HPLC. Peptides were first separated into 80 fractions with a gradient of 2 to 60% acetonitrile in 10 mM ammonium bicarbonate pH10 for 80 min. Next, the peptides were combined into 18 fractions and dried by vacuum centrifuging.

### LC-MS/MS analysis

Three parallel analyses were performed for each fraction. The enriched peptides were analyzed by Q Exactive™ hybrid quadrupole-Orbitrap (Thermo Fisher Scientific), briefly dissolved in 0.1% formic acid (FA) and directly loaded onto an analytical reversed phase analytical column (Acclaim PepMap RSLC, Thermo Fisher Scientific) with a pre-column (Acclaim PepMap 100, Thermo Fisher Scientific).The gradient comprised an increase from a 5% solvent buffer to a 25% one (0.1% FA in 98% ACN) for 26 min, from 25 to 40% for 8 min, climbing to 80% in 3 min, and then remaining at 80% for the last 3 min.

The resulting peptides were subjected to an NSI source, followed by tandem mass spectrometry (MS/MS) in Q Exactive™ (Thermo Fisher Scientific), coupled online to the UPLC. Intact peptides were detected in the Orbitrap at a resolution of 70,000. Peptides were selected for MS/MS using a NCE setting of 28, and ion fragments were detected in the Orbitrap at a resolution of 17,500. A data-dependent procedure that alternated between one MS scan followed by 20 MS/MS scans was applied for the top 20 precursor ions above a threshold ion count of 1E4 in the MS survey scan, with 30.0 s of dynamic exclusion. The electrospray voltage applied was 2.0 kV. Automatic gain control (AGC) was used to prevent an overfilling of the ion trap; 5E4 ions were accumulated for generation of MS/MS spectra. For MS scans, the m/z scan range was 350 to 1800, and the fixed first mass was set as 100 m/z.

### Database search

The resulting MS/MS data were processed using the Mascot search engine (v.2.3.0). Tandem mass spectra were searched against the SwissProt Green Plant database. Trypsin/P was specified as a cleavage enzyme allowing up to 2 missing cleavages. Mass error was set to 10 ppm for precursor ions and 0.02 Da for fragment ions. Carbamidomethyl on Cys, were specified as fixed modification and oxidation on Met was specified as variable modifications. For protein quantification methods, TMT 6-plex was selected in the Mascot. FDR was adjusted to < 1%, and the peptide ion score was set to ≥ 20.

### Bioinformatics analysis

The Gene Ontology (GO) annotation proteome was derived from the UniProt-GOA database, and the proteins were classified by Gene Ontology annotation based on three categories: biological process, cellular component and molecular function. The domain functional descriptions of Up-regulated proteins were annotated by InterProScan (a sequence analysis application) based on protein sequence alignment method. The GO and domains with a corrected *p*-value < 0.05 were considered significant. Wolfpsort was used to predict subcellular localization of the up-regulated proteins. The protein-protein interaction network was obtained from the String database and the interactions between proteins were performed using Cytoscape software (3.4.0) [25]. The Venn diagram was designed by Venny 2.1.0.

### Assay of enzyme activities

200 mg of leaves with three biological replicates were used for the determination of enzyme activities and handled according to the method of corresponding kit. SOD (EC 1.15.1.1), APX (EC 1.11.1.11) and CAT (EC 1.11.1.6) activities were detected according to the method of Mittova et al. [26]. The GPX (EC 1.11.1.9) activity was assayed as described by Drotar et al. [27]. The statistical analyses of the antioxidant enzyme activities were performed by using SAS 9.4. A value of *P* < 0.05 was considered a statistically significant difference.

## Reviewers’ comments

### Reviewer's report 1

George V (Yura) Shpakovski, Russian Academy of Sciences, Russia

## Reviewer comments

The authors have used biochemical and proteomics approaches to investigate proteome of the alpine plant *Rhododendron chrysanthum* Pall., an endangered species with significant ornamental and medicinal value. Since the protein diversity of the alpine tundra species was never studied before and no proteomics data have been reported for *R. chrysanthum* previously, the finding described in this manuscript can be considered novel. In addition, it was shown (by measurements of the activity of antioxidant enzymes and concentration-accumulation of reactive oxygen species [ROS]) that the antioxidant system probably plays a significant role in *R. chrysanthum* stress survival in the wild, supporting the idea that *R. chrysanthum* can serve as a significant resource for investigating stress tolerance in plants. The manuscript describes a large amount of work (1,395 proteins were identified and 705 of them were quantified) and is well written, and the results obtained are interesting and support the conclusions of the work. Owing to high quality scholarly presentation, I am favorably biased to acceptance. Still, the manuscript would greatly benefit from correction by a native speaker to be checked thoroughly for language and style. Here there are a few minor comments and suggestions for some editing and proofreading (underlined):

1) Biochemical and proteomics analysis of antioxidant enzymes reveals the potential stress tolerance in *Rhododendron chrysanthum* Pall. (Title, page 1, lines 1-3)

Author's response: *Thanks for the kind suggestion of Prof. George V (Yura) Shpakovski and we have modified the sentence according to the suggestion.*

2) Many alpine plants defense (overcome?!?) the severe stresses and protect themselves from the oxidative damage by increasing the ratio and activity of antioxidant enzymes. (Abstract, page 1, lines 19-21)

Author's response: *We have corrected this sentence according to the suggestion.*

3) Proteomics method combined with biochemical approach were applied for investigating the oxidation resistance and the potential stress tolerance of *R. chrysanthum* in both protein and molecular level. (Abstract, page 2, lines 1-3)

Author's response: *We have modified the specified sentence.*

4) Specific note: In my opinion, the “stress tolerance” (especially “the potential stress tolerance”) was not studied in the paper.

Author's response: *In our study, the up-regulated expression and higher activities of ROS-scavenging enzymes like Superoxide dismutase, Catalase, Ascorbate peroxidases, and Glutathione peroxidase were observed in the experimental group in comparison to the control. All these enzymes were involved in the coordinated regulation of the homeostasis maintenance under stress and played significant roles in the improvement of stress resistance, so we finally used “the potential stress tolerance” in this paper.*

### Reviewer's report 2

Ramanathan Sowdhamini, Tata Institute of Fundamental Research, India

## Reviewer comments

This manuscript addresses an important issue of how plants respond to abiotic stresses, in general. The authors have approached the question well and performed quality analysis including check for enzyme activity and interacting partners. I would suggest that the manuscript is publishable after they consider few comments as mentioned below. In this paper, the authors have analysed the genes that get upregulated in *Rhododendron chrysanthum* Pall., when subjected to abiotic stresses. This plant grows in the high altitudes in China, where it naturally withstands several abiotic stresses, such as low temperature, low oxygen, cold, UV exposure, poor soil. Using techniques such as tandem mass tags and LC/MS, the genes upregulated between wild and domesticated varieties were compared. Out of 1395 genes studied, 137 of them were identified as upregulated during abiotic stress. Within this set, 14 were noted to be antioxidants through function annotation by consulting Gene Ontology database. In particular, upregulation and higher activities of ROS-scavenging enzymes like Superoxide dismutase, Catalase, Ascorbate peroxidases, and Glutathione peroxidase were observed in the experimental group (subject to abiotic stress) in comparison to the control. These enzymes, in turn, were recorded to engage in protein-protein interactions with other proteins, several of those are upregulated during the given stress. Cellular localizations of these proteins were mostly within cytosol and chloroplast. It is a nice piece of work and I would recommend acceptance of this manuscript in Biology Direct.

1) The list of upregulated genes, function annotation and Arabidopsis orthologs could be provided as Additional files.

Author's response: *We very much appreciate the overall comments of Prof. Sowdhamini. As Prof. Sowdhamini suggested that the list of all up-regulated proteins and GO functional classification of these proteins were provided as* *Additional file* *1* *: Table and Additional file* *2* *: Table.*

2) It is not clear why the authors did not choose transcriptomics to follow the differential gene expression patterns. It will be interesting to also consider the tissue localization of these genes through either direct transcriptome data or derived from orthologs in Arabidopsis thaliana and consulting Plant Ontology databases as well.

Author's response: *Thanks for Prof. Sowdhamini’s valuable comments. We found that we still have many inadequacies in our current work. In future study, we will perform transcriptome and phosphorylation analyses for the further investigation of the stress tolerance mechanisms.*

3) Certain abbreviations, like EG and CG (standing for experimental group and control group, respectively) are not clear. These are defined much later in Methods.

Author's response: *We have made correction according to the comment.*

4) This sentence is not very clear: “Among these, 5 up-regulation proteins were 1 shaped as rhombus and the weight of 2 protein was classified by color in this network.” If it is a technical matter, it could be moved to Figure legend.

Author's response: *We have re-written this part according to the suggestion*.

## Additional files

Additional file 1: GO functional classification of all up-regulated proteins. (XLSX 67 kb)

Additional file 2: All up-regulated proteins. (XLSX 30 kb)

## Abbreviations

ACN: Acetonitrile
APX: Ascorbate peroxidase
CAT: Catalase
DTT: Dithiothreitol
FA: Formic acid
GO: Gene ontology
GPX: Glutathione peroxidase
SOD: Superoxide dismutase
TMT labeling: Tandem mass tags labeling
